# Computational Study of Sensitivity Enhancement in Surface Plasmon Resonance (SPR) Biosensors by Using the Inclusion of the Core-Shell for Biomaterial Sample Detection

**DOI:** 10.3390/bios8030075

**Published:** 2018-08-07

**Authors:** Widayanti, Kamsul Abraha, Agung Bambang Setio Utomo

**Affiliations:** 1 Department of Physics, Universitas Islam Negeri (UIN) Sunan Kalijaga, Yogyakarta 55281, Indonesia; widayan76@gmail.com; 2 Department of Physics, Universitas Gadjah Mada, Sekip Utara, Yogyakarta 55281, Indonesia; agungbambang@ugm.ac.id

**Keywords:** biomaterial detection, SPR-based biosensors, computational study, Fe_3_O_4_@Au core-shell

## Abstract

A theoretical analysis and computational study of biomaterial sample detection with surface plasmon resonance (SPR) phenomenon spectroscopy are presented in this work with the objective of achieving more sensitive detection. In this paper, a Fe_3_O_4_@Au core-shell, a nanocomposite spherical nanoparticle consisting of a spherical Fe_3_O_4_ core covered by an Au shell, was used as an active material for biomaterial sample detection, such as for blood plasma, haemoglobin (Hb) cytoplasm and lecithin, with a wavelength of 632.8 nm. We present the detection amplification technique through an attenuated total reflection (ATR) spectrum in the Kretschmann configuration. The system consists of a four-layer material, i.e., prism/Ag/Fe_3_O_4_@Au + biomaterial sample/air. The effective permittivity determination of the core-shell nanoparticle (Fe_3_O_4_@Au) and the composite (Fe_3_O_4_@Au + biomaterial sample) was done by applying the effective medium theory approximation, and the calculation of the reflectivity was carried out by varying the size of the core-shell, volume fraction and biomaterial sample. In this model, the refractive index (RI) of the BK7 prism is 1.51; the RI of the Ag thin film is 0.13455 + 3.98651*i* with a thickness of 40 nm; and the RI of the composite is varied depending on the size of the nanoparticle core-shell and the RI of the biomaterial samples. Our results show that by varying the sizes of the core-shell, volume fraction and the RIs of the biomaterial samples, the dip in the reflectivity (ATR) spectrum is shifted to the larger angle of incident light, and the addition of a core-shell in the conventional SPR-based biosensor leads to the enhancement of the SPR biosensor sensitivity. For a core-shell with a radius *a* = 2.5 nm, the sensitivity increased by 10% for blood plasma detection, 47.72% for Hb cytoplasm detection and by 22.08% for lecithin detection compared to the sensitivity of the conventional SPR-based biosensor without core-shell addition.

## 1. Introduction

Currently, there is increasing interest in the development of magnetic and plasmonic nanoparticles as active materials for biomolecule detection [1]. A new nanoparticle that combines multiple functions or properties has attracted considerable attention because of its revolutionary technology which enables the sensitivity enhancement of surface plasmon resonance (SPR)-based biosensors. The optical sensor based on SPR is one of the most sensitive methods for detecting biomolecules and works by detecting the changes of the material refractive index, having a fast response and real-time, bio-specific interaction analysis as well as being a label-free technique [2]. SPR is a physical process that occurs when the wave vector of the evanescent wave (EW) matches the wave vector of the surface plasmon (SP) under the total internal reflection condition. This resonance condition is expressed as

(1) $$\frac{\omega_{0}}{c}n_{p}\sin\theta_{\mathrm{SPR}}=\frac{\omega_{0}}{c}{\left(\frac{\varepsilon_{m}n_{d}^{2}}{\varepsilon_{m}+n_{d}^{2}}\right)}^{1/2}\;$$

The variable on left hand side is the propagation constant of a light beam incident at a resonance angle $\theta_{\mathrm{SPR}}$ through the light coupling device (prism) of a refractive index, $n_{p}$, while the right-hand is the propagation constant with $\varepsilon_{m}$ as real part of the metal permittivity and $n_{d}$ as the refractive index of a dielectric material or sensing medium. $\omega_{0}$ and c are the light frequency and the speed of the light in a vacuum, respectively. The evanescent wave occurs at the metal–dielectric interface when a *p*-polarized wave passes a prism through a metallic layer into a dielectric media. 

The wave vector of the evanescent wave is a function of refractive indices of the dielectric, metal and analyte, i.e., the sensing medium. Therefore, if there is a local change in the refractive index of the sensing medium near the metal surface, this will in turn lead to a change in the propagation constant of SP and in the angle of incidence of light in order to satisfy the resonance. When applying an SPR biosensor, the Kretschmann geometry [3] of attenuated total reflection (ATR) has been found to be very suitable for this sensing and has become the most widely used geometry in SPR biosensors. Mostly, the metallic layer that is used in SPR biosensor measurement consists of either gold or silver. The first demonstration of SPR-based sensors for bio-sensing was reported in 1983 by Liedberg et al. [4]. Several methods to enhance the sensitivity of SPR biosensors for detecting biomolecules have been explored for the detection of DNA hybridization [5], acetylcholinesterase [6], membrane protein [7] and human blood-group [8]. SPR can also be a potential candidate for bio-sensing other biological properties such as haemoglobin concentration. 

Some research has indicated that the conventional SPR-based biosensor was not capable of sensing a small amount of biomolecules such as DNA, virus or bacteria [9] due to the poor attachment of biomolecules onto the metal surface, and the fact that the low concentrations involved are difficult to detect directly [10]. In addition, the changes in the refractive index of the medium [11] under a thin metal layer are very small. Therefore, the enhancement of sensitivity for detecting small biomolecules can be developed by several approaches, such as by adding a nanoparticle core-shell as the active material in the conventional SPR-based biosensor. Comparing with a nanoparticle with a spherical shape, the inclusion of a core-shell aims to avoid polar resonance [12] and to obtain the plasmonic wavelength by varying the radius of the core and the thickness of the shell. A core-shell was said to be a unique material since it is a combination of magnetic and plasmonic materials which have different optical properties to the core and the shell. Some studies have observed, either experimentally or theoretically, the optical properties of the core-shell when included in SPR-based biosensors. It is observed that the optical response or resonance spectrum of the core-shell depends on the size of the core and the thickness of the shell. Hence, the core-shell can be used to tune the plasmonic wavelength [13], e.g., for AgSiO_2_ [12], TiO_2_@Au, TiO_2_@Ag [14] and Fe_3_O_4_@Au [15]. The study of the optical response of an Fe_3_O_4_@Au core-shell was performed by varying the radius of Fe_3_O_4_ and the thickness of Au. There was a shift in the resonance spectrum due to the changes in the size of the core and the shell. The core Fe_3_O_4_ can make the biomolecule attachment easier with its magnetic properties, while the shell Au exhibits nontoxicity and compatible properties. Furthermore, the performance of the SPR-based biosensor can be enhanced by using the nanoparticle core-shell Fe_3_O_4_@Au rather than using only Fe_3_O_4_ or only Au. The presence of Fe_3_O_4_@Au is also capable of enhancing the immobilization of biomolecules such as haemoglobin [16], as well as detecting antibody IgG [17] and detecting the DNA of chum salmon [18]. The inclusion of Fe_3_O_4_@Au in the SPR-based biosensor was performed for the enhancement of the detection of thrombin [19] and the protein concentration of interleukin IL17 [20]. Detection of haemoglobin concentration has been explored by SPR-based biosensors for three wavelengths (401.5 nm, 589.3 nm and 706.5 nm) with haemoglobin concentrations varying between 0 and 140 g/L [21].

Due to the coating of the Au shell for the magnetic core, which protected it from oxidation and aggregation, the stabilization of the core-shell (Fe_3_O_4_@Au) was obviously enhanced. In addition, the current SPR technology has a number of advantages; i.e., a high processing sensitivity and selectivity, being non-destructive, a large tunability from the visible into the infrared (IR) spectrum region, label-free analysis and being capable of real-time monitoring. SPR is a kind of electromagnetic resonance that exists when there is an interface between the metal and dielectric. This system has been used to sense various biomolecules [22,23]. This method is very sensitive to size, shape and the refractive index of the surrounding medium that keeps contact with the thin metal layer. When the biomolecule comes into contact with the metal thin film, it is adsorbed onto its surface and hence increases the refractive index at the interface, resulting in a change of their resonance angle.

In this paper, we have been investigating the ATR spectrum of four-multilayer biosensors based on an SPR system with Fe_3_O_4_@Au core-shell addition to detect biology samples that play roles in the interaction of light and the tissue of the body, such as blood plasma, haemoglobin (Hb) cytoplasm and lecithin. The magnetic properties of Fe_3_O_4_@Au are not utilized in this work because we were not applied the external magnetic field. We concern at the Fe_3_O_4_@Au properties such as its dispersivity, easily to its fabrication and synthesis and biocompatible. The refractive indices of those biomaterial samples have been measured by total internal reflection [24]. The blood plasma delivers oxygen and nutrients to the cells of the organs of the body and also transports waste products to be excreted. Hb cytoplasm is the functional molecule in the erythrocytes, increasing oxygen carrying capacity. Lecithin is the fundamental component of the membrane lipid of cells. The different effective permittivity of Fe_3_O_4_@Au + biomaterial composites leads to a change in the SPR resonance angle. This study was focused on the effects of the size of the core radius and shell thickness on the effective permittivity of Fe_3_O_4_@Au by computational approximation. Also, we studied the effects of the volume fraction and the size of the core-shell on the composite effective permittivity and on the reflectivity of the SPR-based biosensor for biomaterial sample detection. Then, the enhancement of the sensitivity of SPR configuration was estimated.

## 2. Materials and Methods

### 2.1. Kretschmann Configuration with Four Layers

**Figure 1:**
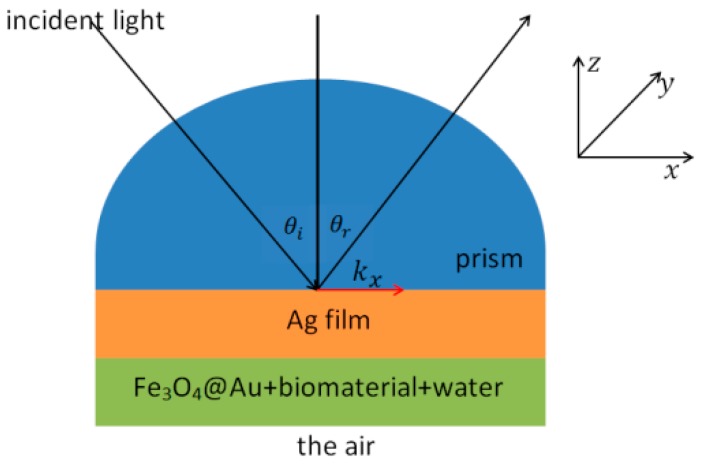
Kretschmann configuration for a surface plasmon resonance (SPR)-based biosensor with the inclusion of Fe_3_O_4_@Au core-shell.

Here, we apply the analytical and computational approximation to calculate reflectivity in the attenuated total reflection (ATR) method and determined the effective permittivity of the composite (the mixture of Fe_3_O_4_@Au and biomaterial embedded in water). In this study, we used the Kretschmann configuration [3] with four layers. I.e., prism/Ag/composite/air, shown in Figure 1. The angle $\theta_{i}$ and $\theta_{r}$ are the incident and the reflection angle, respectively, $k_{x}$ is the wave vector component along *x*-axis, and *d* is the thickness of each layer.

**Figure 2:**
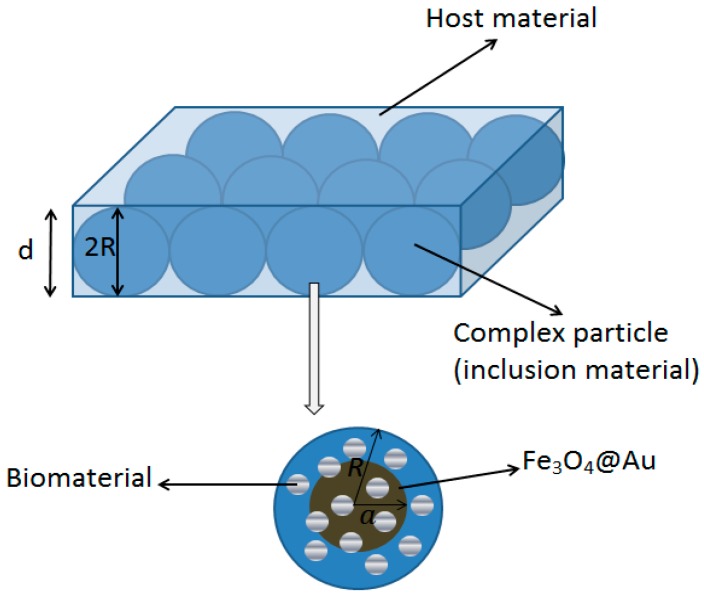
The composite model contains the complex particle (inclusion material) and the host material.

Figure 2 shows the model of the composite layer containing the inclusion material (Fe_3_O_4_@Au + biomaterial samples) and the host material (water). The inclusion material consists of the scattered grain material (Fe_3_O_4_@Au) and the interfacial shell material (biomaterial sample).

In this SPR configuration, the refractive index of the BK7 glass prism is 1.510, the wavelength of the electromagnetic wave is 632.8 nm, the complex refractive index of silver 0.13455 + 3.98651*i* [25], and the refractive index of biomaterial sample is varied. The refractive index of water and air is 1.33 and 1.0, respectively [9]. The thickness of the Ag film was *d* = 40 nm, and the composite was *d* = 20 nm. The ATR reflectivity *R* is given by the Fresnel equation [26].

(2) $$R={\left|r_{i\;j\;k}\right|}^{2}={\left|\frac{r_{i\;j}+r_{j\;k}e^{2ik_{j\;z}d_{j}}}{1+r_{i\;j}r_{j\;k}e^{2ik_{j\;z}d_{j}}}\right|}^{2}\;$$

with

(3) $$r_{i\;j}=\frac{k_{i}\varepsilon_{j}-k_{j}\varepsilon_{i}}{k_{i}\varepsilon_{j}+k_{j}\varepsilon_{i}}\;$$

where $r_{ij}$ is the surface reflectivity coefficient between medium *i* and medium *j*. $k_{ij}$ is the wave vector component perpendicular to the surface, $k_{x}$ is the wave vector component parallel to the surface, whereas $d_{j}$ and $\varepsilon_{i}$ are, respectively, the *j*-th layer thickness and the *i*-th medium dielectric constant.

### 2.2. The Effective Permittivity of the Spherical Core-Shell

**Figure 3:**
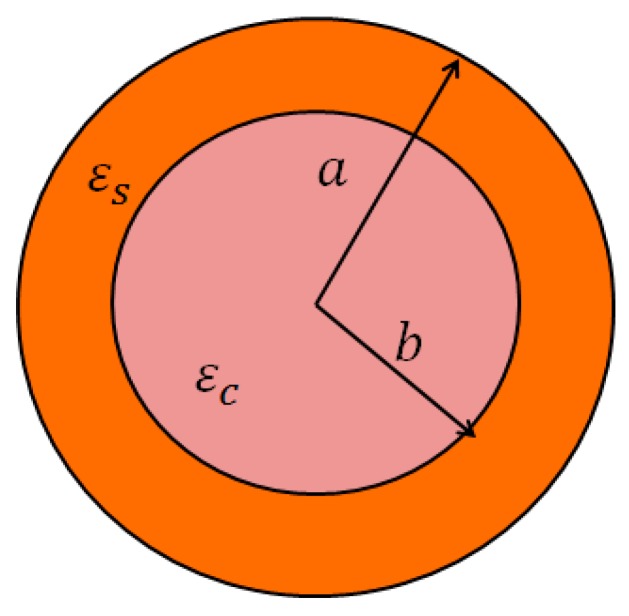
The model of the nanoparticle Fe_3_O_4_@Au core-shell.

Our simulation of the Fe_3_O_4_@Au core-shell is performed on the model as shown in Figure 3. The magnetic nanoparticle core-shell consists of an Fe_3_O_4_ core of radius *b* coated by a metallic Au of thickness (a−b). The dielectric constants of the magnetic nanoparticle and the metallic Au are $\varepsilon_{c}$, and $\varepsilon_{s}$, respectively. The dielectric constants of the materials are dependent on their refractive indices.

The value of complex $\varepsilon_{c}$ is adopted from Schlegel [27] through reflectivity measurement and the Kramers-Kronig relation, and $\varepsilon_{s}$ can be quoted from the work by Johnson and Christy [25]. The effective permittivity $\left(\varepsilon_{\mathrm{eff}}\right)$ of the Fe_3_O_4_@Au core-shell is derived from the internal homogenization for plasmonic and dielectric constituent materials [28], namely

(4) $$\varepsilon_{\mathrm{eff}}=\varepsilon_{s}\frac{a^{3}\left(\varepsilon_{c}+2\varepsilon_{s}\right)+2b^{3}\left(\varepsilon_{c}-\varepsilon_{s}\right)}{a^{3}\left(\varepsilon_{c}+2\varepsilon_{s}\right)-b^{3}\left(\varepsilon_{c}-\varepsilon_{s}\right)}\;$$

### 2.3. The Effective Permittivity of the Composite

The effective permittivity of the composite $\left(\varepsilon_{\mathrm{effc}}\right)$ is calculated by neglecting the correlation between the inclusion material (complex material or Fe_3_O_4_@Au + biomaterial sample) and host material (water) using the Maxwell Garnett formula [29]:

(5) $$(1-F)\frac{\varepsilon_{\mathrm{effc}}-\varepsilon_{m}}{2\varepsilon_{\mathrm{effc}}+\varepsilon_{m}}+F\left(\frac{\varepsilon_{\mathrm{effc}}-\varepsilon_{n}}{\varepsilon_{\mathrm{effc}}+\varepsilon_{n}}\right)=0\;$$

with

(6) $$\varepsilon_{n}=\varepsilon_{1}\frac{\left(2\varepsilon_{1}+\varepsilon_{2}\right)+2\alpha \left(\varepsilon_{2}-\varepsilon_{1}\right)}{\left(2\varepsilon_{1}+\varepsilon_{2}\right)-\alpha \left(\varepsilon_{2}-\varepsilon_{1}\right)}\;$$

where α=(aR)3, a is the radius of Fe_3_O_4_@Au, R is the radius of the complex particle (Fe_3_O_4_@Au + biomaterial sample), F is the volume fraction of the inclusion material to the host material, $\varepsilon_{n}$ is the dielectric constant of the complex particle and $\varepsilon_{m}$ is the dielectric constant of the host material. $\varepsilon_{1}$ is the biomaterial sample dielectric constant as the interfacial shell, while $\varepsilon_{2}$ is the dielectric constant of the scattered grain (Fe_3_O_4_@Au core-shell). In this condition $\varepsilon_{2}=\varepsilon_{\mathrm{eff}}$ in the Equation (4).

### 2.4. Sensitivity from ATR Spectrum

The calculation of the sensitivity of the SPR-based biosensor is written as [30]

(7) $$S=\frac{\Delta \theta_{\mathrm{SPR}}}{\Delta n}\;$$

where $\Delta \theta_{\mathrm{SPR}}$ is the difference in the SPR angle and Δn is the change in refractive index. Whereas the sensitivity enhancement can be obtained from the relation

(8) $$\Delta S=\frac{S_{\mathrm{core}-\mathrm{shell}}-S_{\mathrm{no}\;\mathrm{core}-\mathrm{shell}}}{S_{\mathrm{no}\;\mathrm{core}-\mathrm{shell}}}\times 100\%\;$$

where $S_{\mathrm{core}-\mathrm{shell}}$ is the sensitivity of the conventional system (prism/Ag/biomaterial sample/air), $S_{\mathrm{no}\;\mathrm{core}-\mathrm{shell}}$ is the sensitivity of the proposed system (prism/Ag/Fe_3_O_4_@Au + biomaterial sample/air).

## 3. Results and Discussion

The changes of the radius of the Fe_3_O_4_ core and the thickness of the Au shell lead to changes in the effective permittivity of the Fe_3_O_4_@Au core-shell, while the change of the inclusion material to the host material leads to a change in the effective permittivity of the composite. Therefore, if the complex particle is applied to the SPR-based biosensor system, this change leads to the enhancement of the sensitivity of this biosensor. We can show from the reflectivity spectrum that the resonant angle shifts to the right.

**Figure 4:**
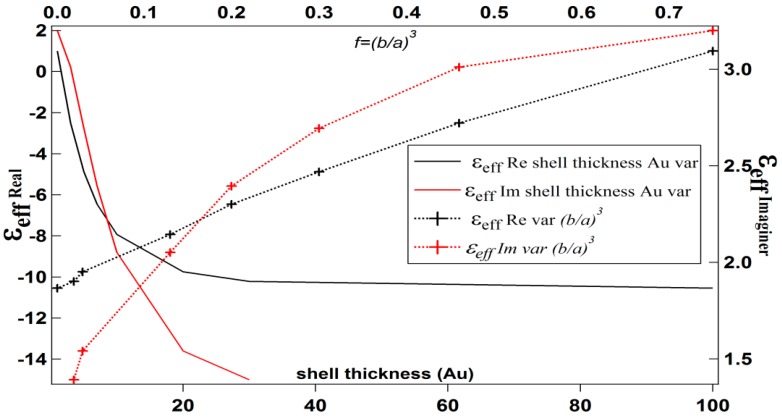
The effective permittivity of a core-shell for variations of shell thickness and $f={\left(b/a\right)}^{3}$.

**Figure 5:**
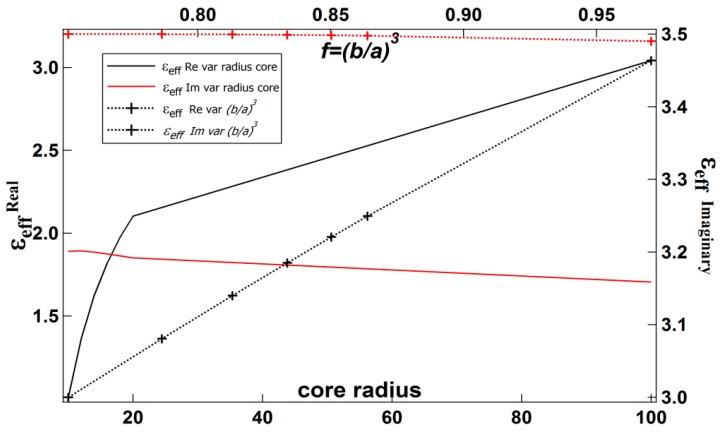
The effective permittivity of a core-shell for variations of the radius of the core and $f={\left(b/a\right)}^{3}$.

**Figure 6:**
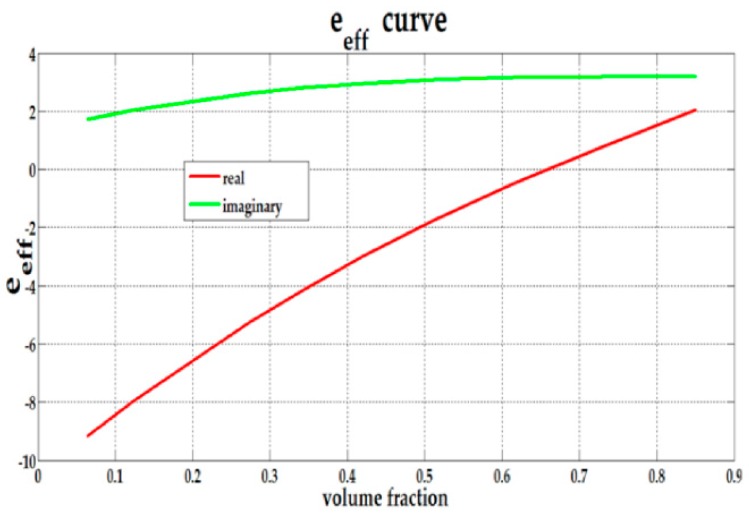
The effective permittivity of a core-shell for variations of $f={\left(b/a\right)}^{3}$.

Table 1 shows the effective permittivity of the core-shell (Equation (4)) for variations in the shell thickness. Table 2 shows the effective permittivity of the core-shell for variations in the core radius, and Table 3 shows the effective permittivity of the core-shell for f=(ba)3 variation. The data in Table 1, Table 2 and Table 3 are presented in Figure 4, Figure 5 and Figure 6, respectively.

Figure 4 shows that the increasing shell thickness for a fixed core radius (10 nm) leads to the decreasing of the real and imaginary part of the core-shell’s effective permittivity. However, the real part tends to be constant at the shell thickness above 30 nm.

Figure 5 shows that increasing the core radius for a fixed shell thickness (1 nm) leads to an increase in the real part of the core-shell’s effective permittivity while the imaginary part tends to be constant.

If the core-shell’s permittivity is viewed only from the $f={\left(b/a\right)}^{3}$ variation, it shows that the increase of $f={\left(b/a\right)}^{3}$ leads to an increase in the real and imaginary parts of the effective permittivity (Figure 6). Different radii of core-shell (*a =* 5 nm) with the same $f={\left(b/a\right)}^{3}$ variation shows the same effective permittivity value. Then, the effective permittivity of the composite can be obtained from Equation (5). 

**Figure 7:**
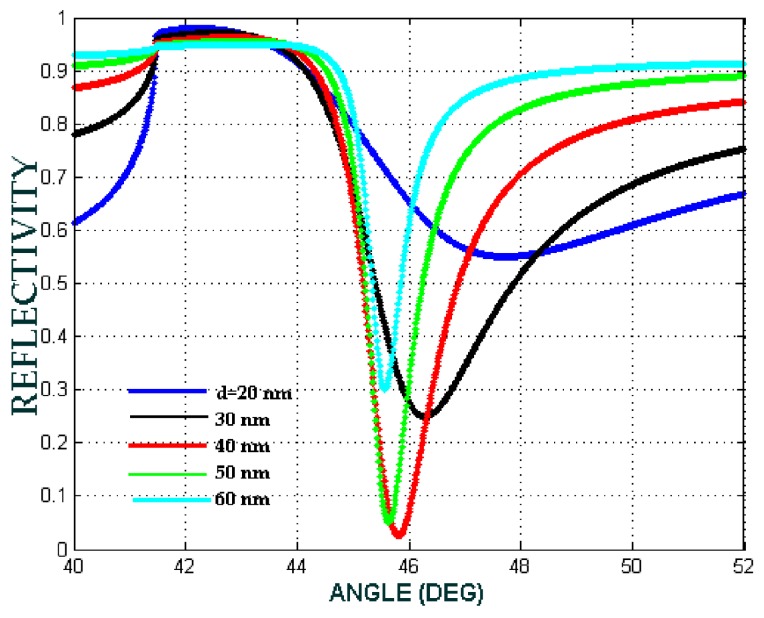
The attenuated total reflection (ATR) spectra for Ag metal thicknesses varying from 20 nm to 60 nm with the SPR biosensor configuration prism/Ag/Fe_3_O_4_@Au + biomaterial sample/air at *a* = 2.5 nm, the biomaterial sample is Hb cytoplasm with RI 1.3800 and *F* = 0.1.

The thicknesses of the Ag metal in this SPR system is the other parameter that must be carefully controlled in order to obtain optimum performance for surface plasmon excitation. Therefore, the choice of the metal thickness is of utmost importance. In the configuration of SPR biosensor is prism/Ag/Fe_3_O_4_@Au + biomaterial sample/air, by varying the Ag thickness, the ATR spectrum shows where the Ag metal film thickness yields the most desirable resonance peak. As the Ag thickness increases (50–60 nm), the depth of the resonance peak decreases. This indicates a reduced coupling efficiency of light into the SP mode on the film. This is due to the fact that the metal begins to act as a reflectance plane when its thickness increases to a point where light cannot couple into the surface charge oscillations that make up the plasmon mode; whereas if the Ag thin film is very thin (20–30 nm), this results in more coupling into the SP mode, but the amplitude of the evanescent wave that penetrated to dielectric materials on the metal layer is higher than the amplitude of the surface plasmon that generated from metal, the sensitivity was reduced. Obviously, from these effects, a compromise must be reached to obtain a satisfactory SPR system. Figure 7 was shows the optimal thickness to support an SPR system determined to lie at 40 nm. From other literature, we get the standard of the thickness of the metal is in the range 40–50 nm. 

**Figure 8:**
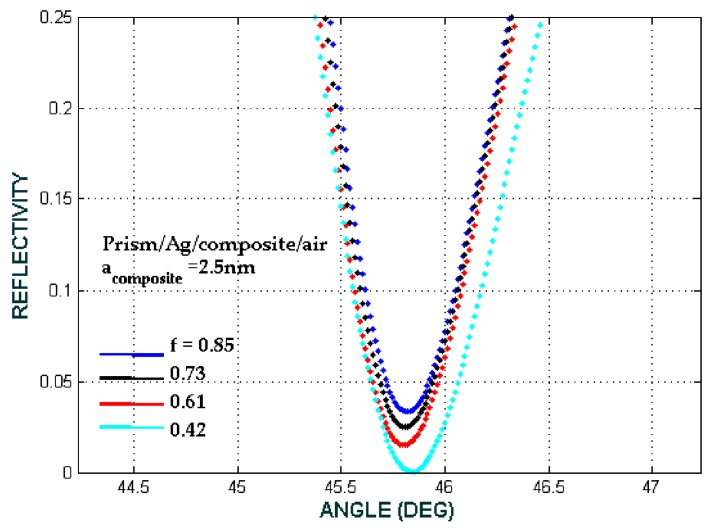
The ATR spectra with variations of $f={\left(b/a\right)}^{3}$ with the SPR biosensor configuration prism/Ag/Fe_3_O_4_@Au/air (*a* = 2.5 nm).

Based on the above results, we can choose the values of the core radii, the shell thickness and the ratio of the core to the core-shell radii $f={\left(b/a\right)}^{3}$ to obtain the desirable effective permittivity of the Fe_3_O_4_@Au core-shell. Figure 8 shows that the desirable effective permittivity is obtained at *f* = 0.85 and *f* = 0.73. For the presented model, we chose the moderate value of *f* = 0.73 for reflectivity calculation because the shell is very thin for *f* = 0.85. 

**Figure 9:**
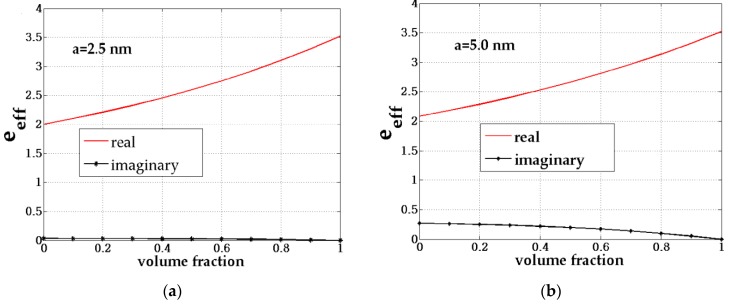
The composite effective permittivity with variations in the volume fraction (*F*) of the composite for the fixed size of the core-shell of (**a**) 2.5 nm and (**b**) 5.0 nm.

**Figure 10:**
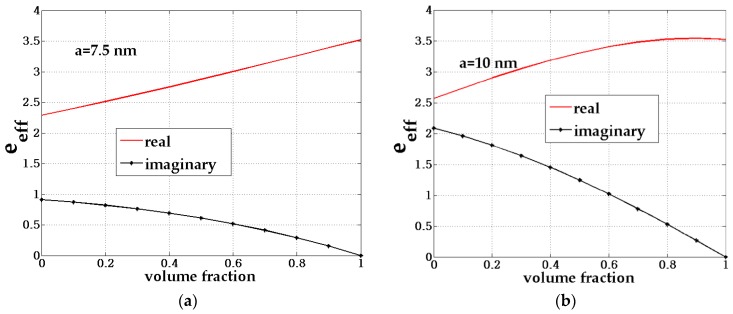
The composite effective permittivity with variations in the volume fraction (*F*) of the composite for the fixed size of the core-shell of (**a**) 7.5 nm and (**b**) 10 nm.

Figure 9 and Figure 10 shows the effective permittivity of the composite (inclusion material) with volume fraction (*F*) variation given by the size variation of the core-shell *a* from 2.5 nm to 10 nm for the chosen fixed $f={\left(b/a\right)}^{3}=0.73$. Here, *F* is the ratio between the amount of the inclusion material to the host material.

**Figure 11:**
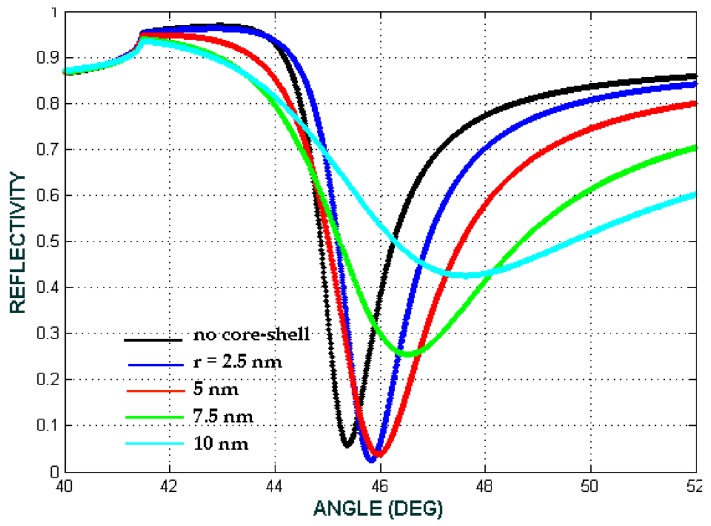
The ATR spectra for the volume fraction of the composite *F* = 0.1. The radii of the core-shell varied from 2.5 nm to 10 nm with a fixed (b/a)^3^ = 0.73 (biomaterial sample is Hb cytoplasm with a refractive index of 1.3871 or the concentration 12.93 mmol L^−1^).

**Figure 12:**
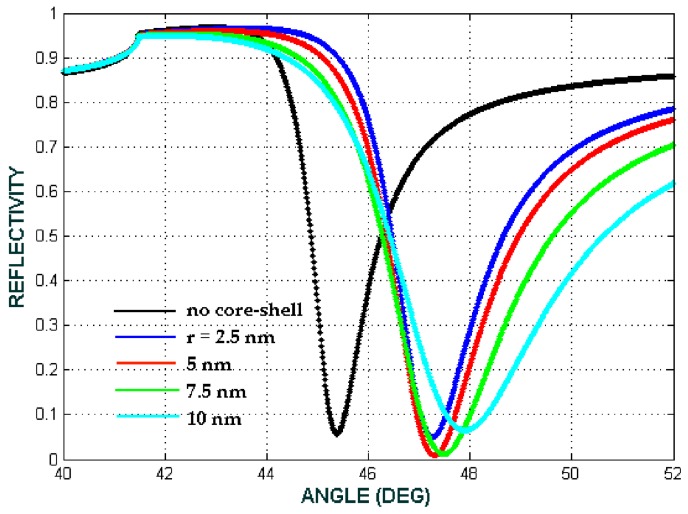
The ATR spectra for the volume fraction of the composite *F* = 0.8. The radius of the core-shell varied from 2.5 nm to 10 nm with a fixed (b/a)^3^ = 0.73 (biomaterial sample is Hb cytoplasma with a refractive index of 1.3871 or the concentration 12.93 mmol L^−1^).

The reflectivity from the SPR-based biosensor consisting of a nanoparticle core-shell is shown in Figure 11 and Figure 12. If the layers only consist of prism/Ag/Hb cytoplasm/air (conventional SPR), the dip of the ATR curve occurs at the incident angle 45.44° (black line). After the composite had been deposited on to the surface of the Ag thin film, the dip of the reflectivity curve was shifted to a larger angle. Referring to Figure 11 for the volume fraction (*F*) = 0.1 and the Fe_3_O_4_@Au radii (*a*) varying from 2.5 nm to 10 nm, the SPR angle was shifted to a larger angle. By increasing the radii of Fe_3_O_4_@Au, the angle of resonance increases as well. It can be seen in Figure 11 that the minimum reflectivity are seen at 45.86° for core-shell radii 2.5 nm, while from Figure 12, for the volume fraction (*F*) = 0.8 and the Fe_3_O_4_@Au radii varying from 2.5 nm to 10 nm, the SPR angle is shifted to a larger angle as well. The figure shows that the minimum reflectivity occurred at 47.40° for core-shell radii of 5 nm and at 47.55° for radii of 7.5 nm. 

**Figure 13:**
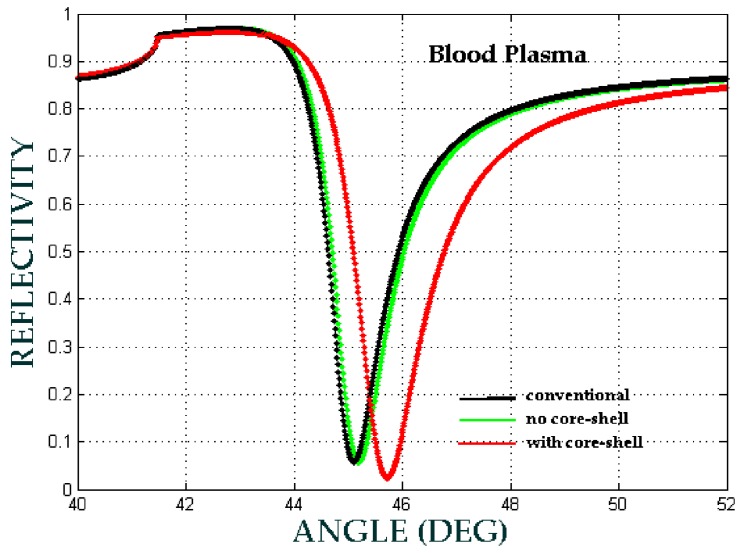
The ATR spectra for different configuration with the volume fraction of the composite *F* = 0.1, the size of the core-shell *a* = 2.5 nm and f  = 0.73. (conventional: Prism/Ag/water/air, no core-shell: Prism/Ag/blood plasma + water/air, with core-shell: Prism/Ag/Fe_3_O_4_@Au+blood plasma + water/air).

**Figure 14:**
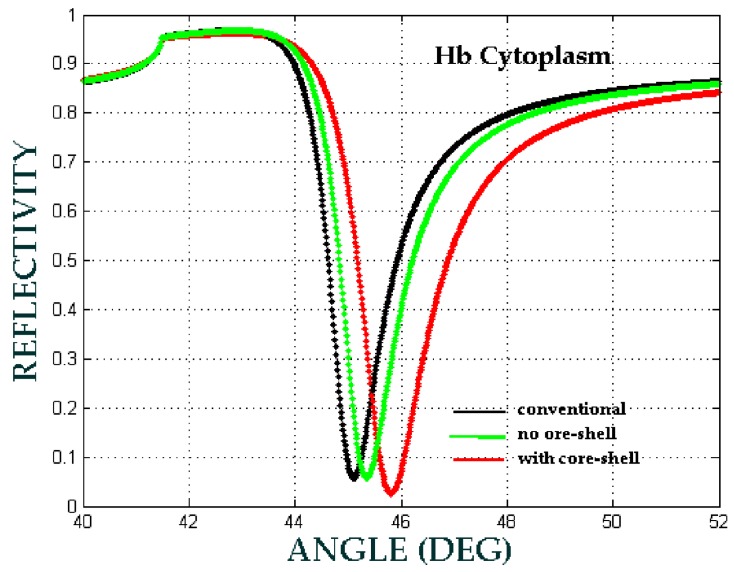
The ATR spectra for different configuration with the volume fraction of the composite *F* = 0.1, the size of the core-shell *a* = 2.5 nm and f  = 0.73. (conventional: Prism/Ag/water/air, no core-shell: Prism/Ag/Hb Cytoplasm + water/air, with core-shell: Prism/Ag/Fe_3_O_4_@Au+Hb cytoplasm + water/air).

**Figure 15:**
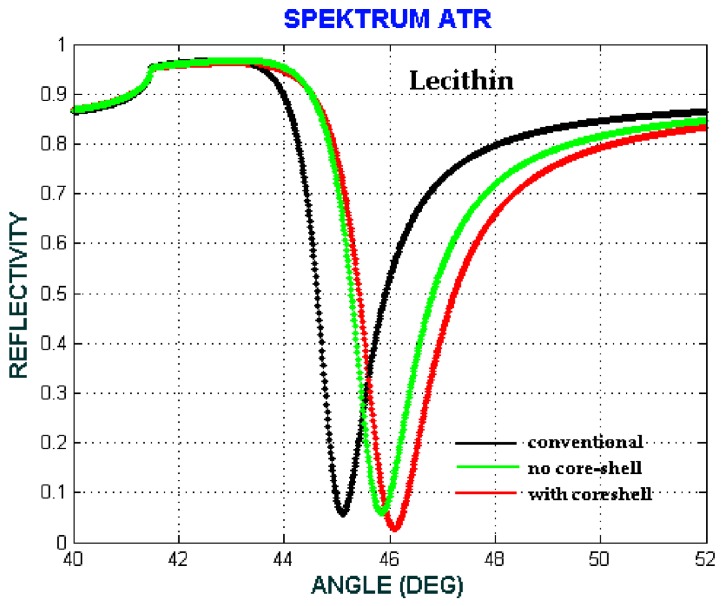
The ATR spectra for different configuration with the volume fraction of the composite *F* = 0.1, the size of the core-shell *a* = 2.5 nm and f  = 0.73. (conventional: Prism/Ag/water/air, no core-shell: Prism/Ag/Lecithin + water/air, with core-shell: Prism/Ag/Fe_3_O_4_@Au + Lecithin + water/air).

In this presented model, biomaterial samples are blood plasma, Hb cytoplasm and lecithin with RIs of 1.3479, 1.3800 and 1.4838, respectively. Figure 13, Figure 14 and Figure 15 show the comparison of the angular shift of the SPR angle for three different configuration respectively.

The angular shift of SPR angle have been showed at Table 4, where $\Delta \theta_{1}$ is the angular shift between no core-shell configuration with the conventional configuration, whereas $\Delta \theta_{2}$ is the angular shift between with core-shell configuration and no core-shell configuration.

We can see that the angular shift for with core-shell configuration is higher than the angular shift for no core-shell configuration. Therefore, we can say that the involvement of Fe3O4@Au core-shell enhance the sensitivity of biomaterial detection.

Therefore, the sensitivity and the sensitivity enhancement can be obtained from Equations (7) and (8) respectively that, the sensitivity enhancement is 10% for blood plasma, 47.72% for Hb cytoplasm and 22.08% for lecithin. The selectivity of the SPR sensor is shown in Figure 13, Figure 14 and Figure 15, in which each biomaterial sample RI value has a unique SPR angle. An increase in RI leads to an increase of the SPR angle. According to these results, the SPR phenomenon sensor with the core-shell addition can be used to selectively and sensitively detect biomaterial samples.

## 4. Conclusions

In summary, we have presented a theoretical analysis and computational study of the effect of the inclusion of a Fe_3_O_4_@Au core-shell to enhance the sensitivity of an SPR-based biosensor through ATR spectra investigation (the magnetic properties of Fe_3_O_4_@Au are not utilized in this work). Our calculations confirm that the property combination of the magnetic and plasmonic materials leads to the enhancement of the SPR-based biosensor sensitivity, which is applied to detect the existence of biomaterial samples such as blood plasma, Hb cytoplasm and lecithin as analytes. By varying the radii of the core (Fe_3_O_4_) and the shell (Au), the refractive index and the permittivity of the core-shell changes and leads to changes in the composite’s (core-shell + biomaterial + water) permittivity. The SPR dip shifted to the right in the reflectivity spectra when the core-shell was added to the composite as the active material. This shift suggests the potential of the core-shell’s inclusion to enhance the sensitivity of SPR biosensors, in this case for sensing biomaterial samples. 

## Figures and Tables

**Table 1:** The effective permittivity of an Fe_3_O_4_@Au core-shell for variations of shell thickness. Imag: imaginary.

*b* (nm)	*a* (nm)	Shell Thickness (nm)	$\;f={\left(b/a\right)}^{3}\;$	$\;\varepsilon_{eff}$ (Real, Imag)
10	11	1	0.75	1.0092, 3.2011
10	13	3	0.46	−2.5021, 3.0123
10	15	5	0.30	−4.8721, 2.6948
10	17	7	0.20	−6.4556, 2.3952
10	20	10	0.13	−7.9297, 2.0516
10	30	20	0.03	−9.7428, 1.5407
10	40	30	0.02	−10.212, 1.3921
10	100	90	0.001	−10.539, 1.2845

**Table 2:** The effective permittivity of an Fe_3_O_4_@Au core-shell for variations of core radius.

*b* (nm)	*a* (nm)	f=(ba)3	$\;\varepsilon_{eff}$ (Real, Imag)
10	11	0.75	1.0092, 3.2011
12	13	0.78	1.3637, 3.2017
14	15	0.81	1.6230, 3.2000
16	17	0.83	1.8209, 3.1975
18	19	0.85	1.9768, 3.1947
20	21	0.86	2.1028, 3.1921
100	101	0.97	3.0427, 3.1587

**Table 3:** The effective permittivity of an Fe_3_O_4_@Au core-shell for variations of $\;f={\left(b/a\right)}^{3}$.

*b* (nm)	*a* (nm)	f=(ba)3	$\;\varepsilon_{eff}$ (Real, Imag)
9.5	10	0.85	2.04008, 3.19339
9.0	10	0.73	0.77837, 3.19889
8.5	10	0.61	−0.49070, 3.16118
8.0	10	0.51	−1.74550, 3.08115
7.5	10	0.42	−2.96636, 2.96227
7.0	10	0.34	−4.13333, 2.81043
6.5	10	0.27	−5.22780, 2.63368

**Table 4:** Angular shit of SPR angle for different biomaterial sample detection.

Biomaterial Sample	$\Delta \theta_{1}(\mathrm{degree})$	$\Delta \theta_{2}(\mathrm{degree})$	$\Delta \theta =\theta_{2}-\theta_{1}(\mathrm{degree})$
Blood Plasma	0.05	0.50	0.55
Hb Cytoplasm	0.21	0.44	0.65
Lecithin	0.71	0.77	0.94

## Supplementary Materials

Supplementary File 1
