# Supplementary material for: Computational Study of Sensitivity Enhancement in Surface Plasmon Resonance (SPR) Biosensors by Using the Inclusion of the Core-Shell for Biomaterial Sample Detection
Source: Biosensors (Basel). 2018 Aug 7;8(3):75. doi: 10.3390/bios8030075 (PMC6164099; doi:10.3390/bios8030075)
Supplement: Supplementary File 1 [file biosensors-08-00075-s001.pdf]

Supplementary Material

```
SOURCE CODE:
MATLAB 2013 a

%Effective permittivity of Composite calculation
%=======================================
clc;clear;
%===================================
x=0.1;
%x=linspace(0,1,11);
em=1.76;      % water (n=1.33)
%ep1=1.9044;      % Hb cytoplasma (n=1.3800)
%ep1=1.8168;    %n=1.3479 Blood Plasma
ep1=2.20166;   %n=1.4838  Lecithin

ep2=0.77837+3.19889*i;    %Fe3O4@Au (f=0.73)
%ep2=2.04008+3.19339*i;
%ep2=-0.49070+3.16118*i;
%ep2=-2.9666+2.6227*i;
r0=2.50d-9;
R=10.0d-9;
alpha=(r0/R)^3;
p=2*ep1+ep2+2*alpha*(ep2-ep1);  %ec (complex particle)
q=2*ep1+ep2-alpha*(ep2-ep1);    %
ec=ep1*(p/q);
%----------rumus abc
a=2;
b=-((2*em)+(ec)-(x.*3*em)+(x.*ec));
c=-(em*ec);
d=sqrt(b.^2-4*a*c);

e_eff1=(b.^1+(sqrt(b.^2-4*a*c)))/2*a;
e_eff2=(b-(sqrt(b.^2-4*a*c)))/2*a;

%ef1=-b/2*a;
%ef2=d/2*a;

n_eff1=sqrt(e_eff1);
n_eff2=sqrt(e_eff2);

ef1r=real(e_eff1);
ef1i=imag(e_eff1);

ef2r=real(e_eff2);
ef2i=imag(e_eff2);

ef1=e_eff1';
ef2=e_eff2';
nf1=n_eff1;
nf2=n_eff2;


sum (e_eff1)
```

```
%plot(x,ef1r,'r',x,ef1i,'g');
%grid on
%xlabel('(b/a)^3');
%ylabel('e_e_f_f');
%title('e_e_f_f curve')
```

%===========ATR SPECTRUM

```
lamda=633.0d-9;
pi=acos(-1.0d0);
p=sqrt(-1);
% ----- Prisma BK7 633 nm -----
en(1)=1.51d0;
ek(1)=0.0d0;
n(1)=en(1)+ek(1)*p;
er(1)=en(1)^2-ek(1)^2;
ei(1)=2.0d0*en(1)*ek(1);
e(1)=er(1)+ei(1)*p;

% ----- Lapisan Tipis Logam Perak(Ag) 633 nm -----
en(2)=0.13455d0;
ek(2)=3.98651d0;
n(2)=en(2)+ek(2)*p;
er(2)=en(2)^2-ek(2)^2;
ei(2)=2.0d0*en(2)*ek(2);
e(2)=er(2)+ei(2)*p;
% ---- Ketebalan Lapisan Tipis Logam perak (meter) -----
d(2)=40.0d-9;


%------ Lapisan Hb----------
en(3)=1.5034d0;
ek(3)=0.0009d0;
n(3)=en(3)+ek(3)*p;
er(3)=en(3)^2-ek(3)^2;
ei(3)=2.0d0*en(3)*ek(3);
%e(3)=1.7689 % water
%e(3)=1.9044+0.0*p;  % n=1.3800 Hb cytoplasma
%e(3)=1.8168+0.0*p;%n=1.3479 Blood Plasma
e(3)=2.20166+0.0*p; %n=1.4838  Lecithin
%===========variasi indeks bias Hb (konsentrasi Hb)================
%e(3)=1.7902+0.0*p;
%e(3)=1.7929+0.0*p;
%e(3)=1.7956+0.0*p;
%e(3)=1.8010+0.0*p;
%e(3)=1.8036+0.0*p;
%e(3)=1.8063+0.0*p;
%e(3)=1.8117+0.0*p;
%e(3)=1.81440*p;
%e(3)=1.8198+0.0*p;
% ----- Ketebalan Hb-----
d(3)= 20.0d-9;
```

```
%--------lapisan Fe3O4+Hb+Water
en(4)=1.5034d0;
ek(4)=0.0009d0;
n(4)=en(4)+ek(4)*p;
er(4)=en(4)^2-ek(4)^2;
ei(4)=2.0d0*en(4)*ek(4);
%e(4)=2.1096+0.0166*p;
% ----- Ketebalan komposit -----
d(4)= 20.0d-9;
%--------lapisan Fe3O4@Au
%e(4)=2.04008+3.1933*p;
%e(4)=0.77837+3.19889*p;
%e(4)=-0.4070+3.16118*p;
%e(4)=-2.96636+2.96227*p;
% ----- Ketebalan komposit -----
d(4)= 20.0d-9;
% ----- Lapisan komposit Fe3O4@Au+Hb+Water-----
%====Hb=1.779======
%e(5)=3.1000+0.0128*p;% 2.5 nm F=0.8    untuk (b/a=18/20=0.73)
%e(5)=3.1361+0.098*p;% 5 nm F=0.8
%e(5)=3.2598+0.2912*p;% 7.5 nm F=0.8
%e(5)=3.5236+0.528*p;%10 nm F=0.8 Eff1
%e(5)=4.6896-11.3446*p;%20 nm F=0.8 Eff2 (tidak terjadi)
%=====Hb=1.7956=========
%e(5)=3.1042+0.0128*p;% 2.5 nm F=0.8    untuk (b/a=18/20=0.73)
%e(5)=3.1398+0.098*p;% 5 nm F=0.8
%e(5)=3.2613+0.2911*p;% 7.5 nm F=0.8
%e(5)=3.5236+0.528*p;%10 nm F=0.8 Eff1
%===========Hb=1.8198=========
%=====Hb=1.7956=========
%e(5)=3.1103+0.0127*p;% 2.5 nm F=0.8    untuk (b/a=18/20=0.73)
%e(5)=3.1442+0.0978*p;% 5 nm F=0.8
%e(5)=3.2636+0.2908*p;% 7.5 nm F=0.8
%e(5)=3.5236+0.528*p;% 10 nm F=0.8

%ep2=2.040082+3.193385i       %(20/19)
%ep2=0.778373+3.19889i    %Fe3O4@Au (20/18)
%ep2=-0.49070+3.161178i       %(20/17)
%ep2=-1.7455+3.081151i       %(20/16)
%ep2=-4.1333+2.81043i       %(20/14)
%ep2=-7.2926+2.0516i     %(20/10)
%============utk r=5 nm=============
%e(5)=2.2022+0.2638*p;   % Blood plasma pada F=0.1
%e(5)=2.2579+0.2662*p;   % Hb cytoplasma
%e(5)=2.3917+0.2684*p;   % lecithin
%============utk r=2.5 nm=============
%e(5)=2.1256+0.0331*p;   % Blood plasma pada F=0.1
%e(5)=2.18124+0.0334*p;   % Hb cytoplasma
e(5)=2.3567+0.0337*p;   % lecithin


%e(5)=3.1437+0.0978*p;   % Blood plasma pada F=0.8
%e(5)=3.1641+0.0965*p;   % Hb cytoplasma
%e(5)=3.2111+0.0921*p;   % lecithin
%=========Hb cytoplasma variasi r F=0.1
%e(5)=2.18124+0.0334*p;   % r=2.5
```

```
%e(5)=2.24785+0.2658*p;    % r=5 nm
%e(5)=2.4328+0.8777*p;    % r=7.5
%e(5)=2.7374+1.9569*p;    % r=10
%=========Hb cytoplasma variasi r F=0.8
%e(5)=3.13061+0.0125*p;    % r=2.5
%e(5)=3.16043+0.0967*p;    % r=5 nm
%e(5)=3.27118+0.2898*p;    % r=7.5
%e(5)=3.5235+0.5287*p;    % r=10
%=========Hb cytopasma variasi f=b/a pada r=2.5 nm ada F=0.1
%e(5)=2.1861104+0.02347*p;    % f=0.85
%e(5)=2.18124+0.0334*p;    % r=0.73
%e(5)=2.17758+0.04916*p;    % r=0.61
%e(5)=2.20040+0.10185*p;    % r=0.42
% ----- Ketebalan komposit -----
d(5)= 20.0d-9;    %d=2D atau 2R

% ----- lapisan 6 udara -----
en(6)=1.0d0;
ek(6)=0.0d0;
n(6)=en(6)+ek(6)*p;
er(6)=en(6)^2-ek(6)^2;
ei(6)=2.0d0*en(6)*ek(6);
e(6)=er(6)+ei(6)*p;

% ----- Scan Sudut Datang -----
ang0=40.0d0;
ang1=52.0d0;

disp(' ');
disp('---------------------------------------------');
disp('Sudut datang | Reflektansi 4 | Reflektansi 5'  );
disp('---------------------------------------------');
for  i=1:1001
   theta=(ang0+(i-1)/1000.0d0*(ang1-ang0))/180.0d0*pi;
   theta_pi=theta/pi*180.0d0;
% ----- Reflektansi -----
  for j=1:6
      kz(j)=(((2.0d0*pi)/lamda))*sqrt(e(j)-e(1)*sin(theta)^2);
   end
  r12=((kz(1)/e(1))-(kz(2)/e(2)))/((kz(1)/e(1))+(kz(2)/e(2)));
  r23=((kz(2)/e(2))-(kz(3)/e(3)))/((kz(2)/e(2))+(kz(3)/e(3)));
  r24=((kz(2)/e(2))-(kz(4)/e(4)))/((kz(2)/e(2))+(kz(4)/e(4)));
  r25=((kz(2)/e(2))-(kz(5)/e(5)))/((kz(2)/e(2))+(kz(5)/e(5)));
  r26=((kz(2)/e(2))-(kz(6)/e(6)))/((kz(2)/e(2))+(kz(6)/e(6)));
  r36=((kz(3)/e(3))-(kz(6)/e(6)))/((kz(3)/e(3))+(kz(6)/e(6)));
  r46=((kz(4)/e(4))-(kz(6)/e(6)))/((kz(4)/e(4))+(kz(6)/e(6)));
  r56=((kz(5)/e(5))-(kz(6)/e(6)))/((kz(5)/e(5))+(kz(6)/e(6)));


r236=(r23+(r36*exp(p*2.0d0*kz(3)*d(3))))/(1+(r23*r36*exp(p*2.0d0*kz(3)*d(3)))
);

r246=(r24+(r46*exp(p*2.0d0*kz(4)*d(4))))/(1+(r24*r46*exp(p*2.0d0*kz(4)*d(4)))
);
```

```
r256=(r25+(r56*exp(p*2.0d0*kz(5)*d(5))))/(1+(r25*r56*exp(p*2.0d0*kz(5)*d(5)))
);
  % ----- Reflektansi tiga Layer P/Ag/Air-----

r1=((r12+(r26*exp(p*2.0d0*kz(2)*d(2))))/(1+(r12*r26*exp(p*2.0d0*kz(2)*d(2))))
);
   r1c=conj(r1);
    ref1=r1*r1c;
  %--------reflektansi eempat layer--------
            %--------P/Ag/Hb/Air--------

r2=((r12+(r236*exp(p*2.0d0*kz(2)*d(2))))/(1+(r12*r236*exp(p*2.0d0*kz(2)*d(2))
)));
   r2c=conj(r2);
    ref2=r2*r2c;
             %-------P/Ag/Fe3O4@Au/Air--------

r3=((r12+(r246*exp(p*2.0d0*kz(2)*d(2))))/(1+(r12*r246*exp(p*2.0d0*kz(2)*d(2))
)));
   r3c=conj(r3);
    ref3=r3*r3c;

             %-------P/Ag/Fe3O4@Au+Hb+Water/Air

r4=((r12+(r256*exp(p*2.0d0*kz(2)*d(2))))/(1+(r12*r256*exp(p*2.0d0*kz(2)*d(2))
)));
   r4c=conj(r4);
    ref4=r4*r4c;

 %fprintf('%10.2f   |  %10.5f   | %10.5f \n',theta_pi,ref2,ref3,ref4);
 fprintf('%10.2f \n',ref4);

 %plot(theta_pi,ref2,'.b',theta_pi,ref3,'.g',theta_pi,ref4,'.k');
 %plot(theta_pi,ref2,'.k',theta_pi,ref4,'.r');
 %plot(theta_pi,ref2, '.k',theta_pi,ref4, 'k--','LineWidth', 24);
 %plot(theta_pi,ref2, '.k',theta_pi,ref4,'k--*','linewidth',0.1);
 plot(theta_pi,ref2, '.g');
 %plot(theta_pi,ref4, '.r');
 %plot(theta_pi,ref4,'--ko','linewidth',1);
      hold on;
      xlim([40 52]);
      ylim([0 1]);
      grid on;
      %set(gca,'color',[.9 0 0.8],'fontname','arial');
      title('SPEKTRUM ATR','color',[0 .1 1],...
         'fontweight','bold','fontsize',14);
      xlabel('ANGLE (DEG)','color',[0 .2 .2],...
         'fontweight','bold','fontsize',12');
      ylabel('REFLECTIVITY','color',[0 .2 .2],...
         'fontweight','bold','fontsize',12);

end
```
